# Facial pressure beneath a cavesson noseband adjusted to different tightness levels during standing and chewing

**DOI:** 10.1111/evj.14451

**Published:** 2024-12-22

**Authors:** Hilary M. Clayton, Rachel Murray, Jane M. Williams, Vicki Walker, Mark Fisher, Diane Fisher, Jane Nixon, Russell Mackechnie‐Guire

**Affiliations:** ^1^ Department of Large Animal Clinical Sciences, College of Veterinary Medicine Michigan State University East Lansing Michigan USA; ^2^ Rossdales LLP Suffolk UK; ^3^ Hartpury University Equestrian Performance Research Centre Gloucester UK; ^4^ Woolcroft Equine Services Wisbech UK; ^5^ Buckingham Equine Vets Overton Fields Buckingham UK

**Keywords:** blink rate, eye temperature, horse, mandibular pressure, nasal pressure, noseband tightness

## Abstract

**Background:**

Noseband adjustment should avoid discomfort and allow some jaw movement.

**Objectives:**

To determine pressure beneath a cavesson noseband at five tightness levels during standing and chewing. It was hypothesised that increased noseband tightness is associated with increases in nasal and mandibular pressures while standing and chewing, accompanied by increases in eye temperature and blink rate.

**Study design:**

Experimental.

**Methods:**

Eight highly‐trained dressage horses wore a snaffle bridle with their own bit. Pressure mats over the nasal bones and beneath the mandibular rami recorded sub‐noseband pressures (50 Hz) for five tightness levels (2.0, 1.5, 1.0, 0.5, 0.0 finger‐equivalents from loosest to tightest) measured using a taper gauge during quiet standing and chewing a treat. Eye temperature and blink rate were recorded synchronously. Data were analysed using Friedmans two‐way ANOVA with Wilcoxon post hoc tests and Bonferroni adjustment for repeated measures. Significance level *p* ≤ 0.01.

**Results:**

During standing, total force increased from (mean ± SD) 5.8 ± 4.4 N (nasal) and 12.3 ± 8.2 N (mandibular) at 2.0 finger‐equivalents to 45.1 ± 24.9 N (nasal) and 70.7 ± 25.7 N (mandibular) at 0.0‐finger‐equivalents. Forces and pressures were higher on the mandibles than nasal bones although differences did not always reach statistical significance. Horses willingly ingested and chewed a treat at all noseband tightness levels generating forces ~100 N and pressure >40 kPa without increases in eye temperature or blink rate that would suggest discomfort. Post hoc tests indicated significantly higher pressure for 0.0 finger‐equivalents than 2.0 finger‐equivalents (*p* < 0.01).

**Main limitations:**

Small sample size. Nosebands always tested from loosest to tightest.

**Conclusions:**

Mandibular pressure exceeded nasal pressure and values at both sites increased with noseband tightness. Horses accepted high noseband pressures when chewing a treat with a cavesson adjusted from 0.0 to 2.0 finger‐equivalents. Blink rate and eye temperature suggest horses were not distressed when chewing at 2.0 to 0.0 finger‐equivalents tightness.

## 1 INTRODUCTION

The equipment used by a rider to communicate with the horse should fit the horse and be adjusted appropriately to fulfil its purpose without compromising welfare. Concerns have been raised about nosebands being tightened to a degree that they may cause high pressures, pain and/or overly restrict movement of the temporomandibular joint (TMJ). An association between oral lesions and noseband type/tightness has been shown.^1^

Traditionally, it is recommended that it should be possible to insert two fingers under a noseband to demonstrate sufficient laxity.^2^ However, finger size is not a standard international unit of measurement and cannot be used objectively so a taper gauge has been developed for this purpose.^3^ This study addresses the need to determine a laxity threshold for nosebands that is effective without compromising equine welfare. One rule of thumb is that horses should be able to ingest and chew a sugar lump after final adjustment of the noseband.^4^ Indeed, willingness to ingest and chew a treat may provide useful information about the horse's sensory experience and has been included in this study as a way to evaluate the restrictive effect of the noseband in relation to the horse's sensory perception. In addition to measuring sub‐noseband forces and pressures, the study reported here uses eye temperature^5^ and blink rate^6^ as indicators of stress or discomfort due to noseband pressure.^3^ These measures were chosen because they are non‐invasive indicators of equine stress^7^, ^8^ and eye temperature has been used previously to detect stress responses.^7^ For example, when the noseband was adjusted so it had no laxity, eye temperature increased.^9^ Eye blink rate is determined by the need for corneal moistening but its frequency changes with emotional and cognitive states. Stress may limit eye closing to avoid loss of visual information.^10^

There have only been a few published studies of noseband pressure on the horse's face. One study reported mean and maximal pressures beneath a cavesson noseband as 26.7 and 32.6 kPa, respectively.^11^ A different study reported maximal pressures over the nasal bone of 46.5 kPa for a standard noseband and 24.5 kPa for a noseband designed to reduce nasal pressure.^12^

The study aims were to: (1) quantify nasal and mandibular pressures simultaneously for a cavesson noseband fitted at five tightness levels as determined by a taper gauge, with the horse standing still and while chewing a treat; and (2) investigate associations between noseband tightness and facial pressure with increases in eye temperature and/or blink rate. It was hypothesised that (1) increased noseband tightness would be associated with increases in mean nasal and mandibular pressures while standing and in mean, minimal and maximal nasal and mandibular pressures when chewing; and (2) higher nasal and mandibular pressures would be accompanied by increases in eye temperature and blink rate that support stress as a response to noseband tightness.

## 2 MATERIALS AND METHODS

### 2.1 Horses

The subjects were eight, privately‐owned dressage horses. All horses were European Warmbloods (4 German, 3 Dutch, 1 British) actively competing at Prix St. Georges and above, with (mean ± SD) height: 1.74 ± 0.07 m; mass: 603 ± 24 kg; age: 12 ± 1 years. Inclusion criteria required horses to be accustomed to being ridden in a cavesson noseband and to have received a full dental evaluation and treatment as needed by a veterinarian (3 horses) or by a British Association of Equine Dental Technicians qualified equine dental technician (5 horses) within 3 months prior to the study.

### 2.2 Bridles and nosebands

Each horse's head conformation was assessed by a Society of Master Saddlers (SMS) qualified bridle fitter. This SMS bridle fitter and two other bridle fitters collectively assembled the same type of snaffle bridle for all horses in the appropriate size and fit for each horse. The bridles had a non‐anatomical mono‐headpiece, two cheek pieces, a throat latch and the horse's own double‐jointed snaffle bit. Holes were punched at 0.5 cm intervals in the bridle straps to allow small adjustments. A cavesson noseband was adjusted with the top of the circumferential strap 2 cm rostral to the facial crest according to industry guidelines from the SMS. The noseband was 3 cm wide with padding 4 mm thick.

### 2.3 Noseband tightness

An International Society for Equitation Science (ISES) taper gauge with marks indicating 1.0 and 2.0 fingers tightness was used. To indicate 1.5 and 0.5 finger tightness, additional lines were added to the gauge at 50% of the distance between the 2.0 and 1.0 and the 1.0 and 0.0 finger tightness reference marks, respectively. The gauge was inserted under the lower edge of the noseband and pushed rostrocaudally between the noseband and the skin on the dorsal midline as far as it would go without dorsally displacing the noseband. Pressure measurements were collected from each horse starting with the loosest setting of 2.0 finger‐equivalents and increasing by half finger‐equivalent increments to 0.0 finger‐equivalents, which was the first setting at which the taper gauge could not be inserted.

### 2.4 Noseband pressures

**FIGURE 1:**
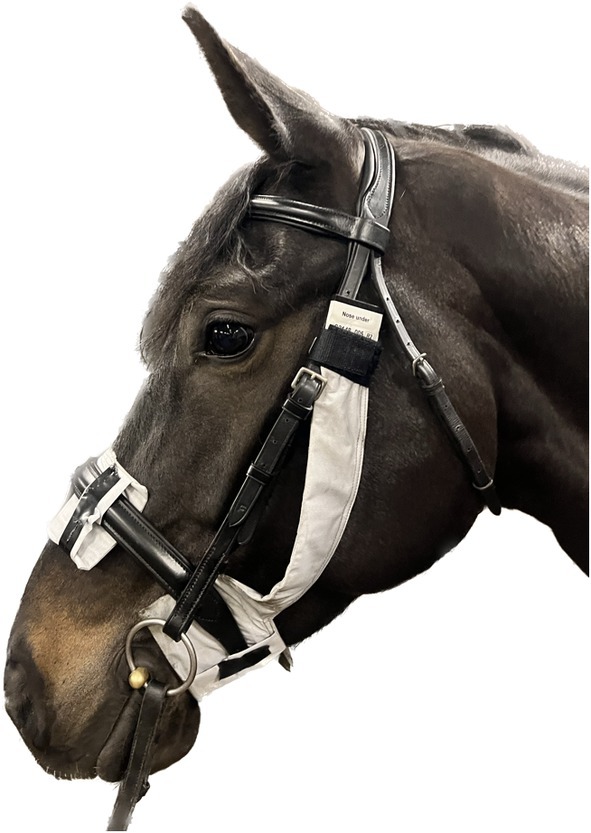
Horse wearing a cavesson noseband with small electronic pressure mats over the nasal and mandibular bones.

Two small pressure mats with 64 sensors in a 16 × 4 configuration and calibrated over a pressure range of 5–240 kPa were used (Pliance, Novel GmbH) (Figure 1). Long cables (CX2022‐ES) from each mat were braided into the horse's mane and connected to the data logger attached to a saddle cloth. The mats were positioned mid‐dorsally over the nasal bones and mid‐ventrally over the mandibular rami (Figure 1). Prior to data collection, they were initialised to zero with the noseband attached and adjusted to greater than 2 finger‐equivalents. A video camera (25 Hz) was hard wired to the laptop, synchronised, and captured within Pliance software, with a synchronised start/stop initiating or terminating data collection.

### 2.5 Eye temperature

An infrared thermal camera (FLIR C3) set perpendicular to and 1 m from the horse's left eye measured eye temperature at the medial canthus.^5^ Thermal recordings were taken before the static assessment, immediately after noseband adjustment to each pre‐determined tension level, and immediately after completion of each 20 s trial. All measurements were taken in an indoor arena. The measuring area was positioned away from the door, all windows (if applicable) were closed, and the area was free from sunlight and draughts. The ambient temperature ranged from 10 to 11°C, the outside temperature was 4.4–8.2°C, and the humidity was 95%–98%.

### 2.6 Blink rate

A 30 Hz video camera 3 m from and perpendicular to the horse's right eye recorded eye blinks during standing and chewing trials. The videos were viewed to identify events, such as noseband tightening, and to count the number of full closures of the right eye during standing and chewing trials.^8^ Repeatability of quantification of blink rate performed by the same researcher taking 5 counts for 3 horses was ±1 blink/min.

### 2.7 Study protocol

Horses walked to the data collection area in an indoor arena at their home facility, then walked in the arena for 5 min before being prepared for the study. A baseline measurement of eye temperature was taken before the bridle and pressure mats were put on. Each trial lasted 20 s.

#### 2.7.1 Standing trials

Horses stood squarely with their neck horizontal. Taking care that the mats did not move, the noseband was adjusted to the required tightness using the taper gauge.^13^ Eye temperature was measured, noseband pressure was recorded for 20 s, then eye temperature was re‐measured. If the horse moved their body, neck or head, the trial was aborted and a new trial started. Only two trials were aborted. The noseband was initially adjusted to 2.0 finger‐equivalents and tightness increased in half‐finger increments in successive trials. After completing the standing trials, the noseband was loosened to greater than 2.0 fingers laxity and eye temperature was re‐measured.

#### 2.7.2 Chewing trials

With the noseband adjusted to 2.0 finger‐equivalents, collection of pressure data started and 2 s later a horse treat was offered (4.5 × 1.7 × 1.7 cm) (Spillers, UK). Data collection continued for 20 s while the horse chewed a single treat and horses were given time to finish eating the treat before tightening the noseband by a half‐finger increment in preparation for the next trial. Eye temperature was measured after each noseband adjustment and at the end of each data collection. After all trials had been completed, the recording equipment was removed and the horse returned to the stable.

### 2.8 Outcome measures in standing trials

The force applied to each pressure sensor was calculated as pressure multiplied by sensor area and total force on the mat was the summation of forces over all loaded sensors. Mean ± SD force–time graphs for the nasal and mandibular mats were plotted and mean nasal and mandibular pressures were calculated over the duration of each trial.

### 2.9 Outcome measures in the chewing horse

During chewing trials, the force–time graphs showed a sinusoidal pattern. Chewing cycles were segmented at successive minima and the following variables were determined:

- Chewing cycle frequency.
- Minimal, maximal and mean nasal and mandibular noseband pressures in each chewing cycle.
- Minimal, maximal and mean total nasal and mandibular forces in each chewing cycle.

### 2.10 Data analysis

A priori sample size (G*Power) was performed using mean and standard deviation for paired differences for peak pressure data collected beneath the noseband in a previous study.^12^ This estimated that for a sample size of 8 horses, the study's power would be 0.90. The Kolmogorov Smirnov test indicated non‐parametric data distribution, therefore Friedmans two‐way ANOVAs with post hoc Wilcoxon signed rank tests were used to compare measures across the five noseband tightness levels. For eye temperature and blink rate, Wilcoxon signed rank tests were used to make comparisons from time of tightening to the end of the test for each tightness level. Kruskal–Wallis and post hoc Mann–Whitney *U* analyses evaluated differences between noseband tightness levels. A further Wilcoxon signed rank test compared blink rate at 2.0 and 0.0 finger‐equivalents. As this study included repeated measures, a Bonferroni adjustment was applied, therefore adjusting the significance level to *p* ≤ 0.01. All tests used IBM SPSS Statistics (version 29).

## 3 RESULTS

### 3.1 Standing trials

**FIGURE 2:**
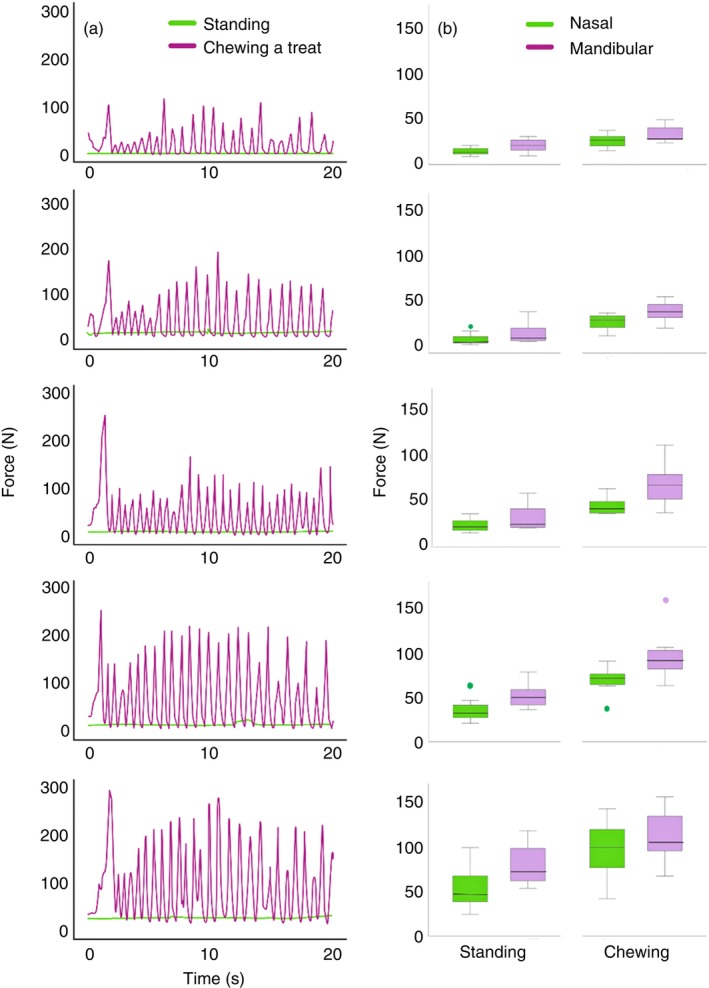
(A) Force–times curves for the nasal mat during 20 s trials for standing and chewing a treat. (B) Nasal and mandibular forces during standing (left two columns) and chewing (right two columns). Central line: median; box: 25th and 75th percentiles; whiskers: maximal and minimal values not considered outliers; dots: outliers. Data are for five noseband tightness levels (top to bottom: 2.0, 1.5, 1.0, 0.5 and 0.0 finger‐equivalents). *N* = 8 horses.

The standing force–time graph (Figure 2) shows consistently low force values with only minor variations during each 20 s trial regardless of noseband tightness. Mean force increased significantly with noseband tightness at the nasal and mandibular sites (Table 1). Post hoc results indicated lower nasal and mandibular force at 2 and 1.5 finger‐equivalents versus 0 finger‐equivalents. During standing, mean force was significantly higher on the mandibles than the nasal bones for all tightness levels (Table 1).

**TABLE 1:** Mean ± SD total force on the nasal and mandibular pressure mats at 5 noseband tightness levels.

Location	Condition	Mean force (N)					*p* value across tightness levels	Post hoc
		2 finger tightness	1.5 finger tightness	1 finger tightness	0.5 finger tightness	0 finger tightness		
Nasal	Standing	5.8 ± 4.4	6.5 ± 7.7	15.3 ± 7.7	26.7 ± 14.4	45.1 ± 24.9	**<0.001**	2 < 0 fingers, *p* = <0.001 1.5 < 0 fingers, *p* = 0.001
Mandibular	Standing	12.3 ± 8.2	13.5 ± 13.1	24.6 ± 14.9	42.3 ± 14.5	70.7 ± 25.7	**<0.001**	2 < 0 fingers, *p* = <0.001 1.5 < 0 fingers, *p* = 0.001
*p* value	Nasal vs. mandibular	**0.01**	**0.01**	**0.01**	**0.01**	**0.01**		
Nasal	Chewing	17.5 ± 8.1	25.3 ± 9.4	37.0 ± 10.5	58.8 ± 16.2	86.6 ± 35.3	**<0.001**	2 < 0 fingers, *p* = <0.001 1.5 < 0 fingers, *p* = 0.007
Mandibular	Chewing	25.4 ± 9.5	37.1 ± 12.3	60.8 ± 25.9	87.4 ± 30.7	102.5 ± 32.4	**<0.001**	2 < 0 fingers, *p* = <0.001
*p* value	Nasal vs. mandibular	**0.01**	**0.01**	**0.01**	0.02	0.02		
*p* value	Nasal Standing vs. chewing	**0.01**	**0.01**	**0.01**	**0.01**	**0.01**		
*p* value	Mandibular Standing vs. chewing	0.02	**0.01**	**0.01**	**0.01**	0.02		

*Note*: Bold values indicate statistically significant differences (*p* ≤ 0.01) between tightness conditions and between nasal and mandibular sites. Shaded boxes: No statistical test performed.

### 3.2 Chewing trials

When chewing a treat, the mandible moved relative to the skull in a somewhat circular motion that generated regular peaks and troughs in the force–time curve (Figure 2A) coinciding with maximal and minimal facial pressures, respectively. When chewing, mean force was higher on the mandibles than the nasal bones when the noseband was adjusted to 2.0, 1.5 and 1.0 finger‐equivalents (Table 1). Nasal and mandibular total chewing forces increased significantly with noseband tightness (Table 1, Figure 2B).

A high, wide force peak occurred as the incisors separated to ingest the treat. Low magnitude force peaks followed as the horses began chewing with a small range of mandibular motion that gradually increased during the middle of the 20 s trial. The force peaks decreased in height or showed an alternating high‐low pattern towards the end of the trial. Mean maximal force increased progressively with noseband tightness (Figure 2A). Mean frequency of the chewing cycles was ~1.25 Hz for all 8 horses and tightness levels.

**FIGURE 3:**
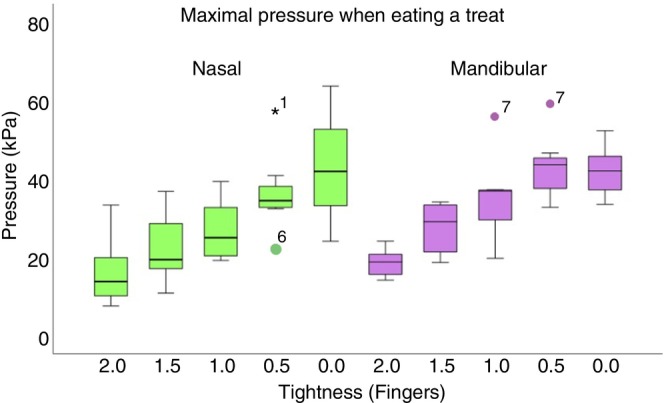
Maximal nasal (green) and mandibular (purple) noseband pressures while chewing a treat with five noseband tightness levels. Central line: median; box: 25th and 75th percentiles; whiskers: maximal and minimal values not considered outliers; dots: outliers.

Overall significance values across tightness levels showed that minimal, maximal and mean chewing pressures increased significantly with noseband tightness at nasal and mandibular sites. The post hoc tests in Table 2 do not show pressure differences between 2 versus 1.5, 1.5 versus 1.0, or 1.0 versus 0.5 finger‐equivalents. During standing, mean mandibular pressures were significantly higher than nasal pressures for all noseband tightness levels. During chewing, mean pressure was significantly higher on the mandibles than nasal bone at 2.0, 1.5 and 1.0 finger‐equivalents (Table 2, Figure 3).

**TABLE 2:** Mean ± SD nasal and mandibular pressures beneath a cavesson noseband adjusted to five tightness levels during standing and chewing.

	Location	Condition	2 finger tightness	1.5 finger tightness	1 finger tightness	0.5 finger tightness	0 finger tightness	*p* value across tightness levels	Post hoc tests
Maximal pressure (kPa)	Nasal	Chewing	16.1 ± 8.3	22.2 ± 8.5	26.8 ± 7.7	36.2 ± 9.8	42.8 ± 13.0	**<0.001**	2 < 0.5 fingers, *p* = 0.003 2 < 0 fingers, *p* = <0.0001 1.5 < 0 fingers, *p* = 0.009
	Mandibular	Chewing	18.6 ± 3.5	27.3 ± 6.7	35.1 ± 11.1	43.0 ± 8.5	41.9 ± 6.4	**<0.001**	2 < 0.5 fingers, *p* = 0.001 2 < 0 fingers, *p* = 0.001
	*p* value	Nasal vs. mandibular	**0.01**	**0.01**	0.04	0.12	0.73		
Minimal pressure (kPa)	Nasal	Chewing	0.1 ± 0.7	0.1 ± 0.1	0.8 ± 0.7	0.9 ± 0.4	2.5 ± 1.9	**<0.001**	2 < 0.5 fingers, *p* = 0.007 2 < 0 fingers, *p* = <0.0001 1.5 < 0 fingers, *p* = 0.001
	Mandibular	Chewing	0.6 ± 1.0	0.6 ± 0.8	2.4 ± 2.3	3.4 ± 2.7	5.2 ± 2.8	**<0.001**	2 < 0.5 fingers, *p* = 0.01 2 < 0 fingers, *p* = <0.0001 1.5 < 0 fingers, *p* = 0.007
	*p* value	Nasal vs. mandibular	0.09	**0.01**	**0.01**	**0.01**	0.04		
Mean pressure (kPa)	Nasal	Standing	0.9 ± 0.6	1.0 ± 1.2	2.3 ± 1.2	4.2 ± 2.2	7.5 ± 3.3	**<0.001**	2 < 0 fingers, *p* = <0.001 1.5 < 0 fingers, *p* = 0.001
	Mandibular	Standing	1.9 ± 1.2	2.1 ± 2.1	3.8 ± 2.3	6.6 ± 2.2	11.1 ± 4.0	**<0.001**	2 < 0 fingers, *p* = <0.001 1.5 < 0 fingers, *p* = 0.001
	*p* value	Standing	**0.01**	**0.01**	**0.01**	**0.01**	**0.01**		
	Nasal	Chewing	2.7 ± 1.2	3.9 ± 1.4	5.7 ± 1.6	9.1 ± 2.5	13.5 ± 5.5	**<0.001**	2 < 0 fingers, *p* = <0.001 1.5 < 0 fingers, *p* = 0.007
	Mandibular	Chewing	3.9 ± 1.4	5.7 ± 1.9	9.5 ± 4.1	13.6 ± 4.8	16.0 ± 5.1	**<0.001**	2 < 0 fingers, *p* = <0.001
	*p* value	Nasal vs. mandibular	**0.01**	**0.01**	**0.01**	0.02	0.02		
	*p* value	Nasal Standing vs. chewing	**0.01**	**0.01**	**0.01**	0.02	0.02		
	*p* value	Mandibular Standing vs. chewing	0.02	**0.01**	**0.01**	**0.01**	0.02		

*Note*: Bold values indicate statistically significant differences (*p* ≤ 0.01) between tightness conditions and nasal versus mandibular pressures. Shaded boxes: No statistical test performed.

### 3.3 Pressure scans

**FIGURE 4:**
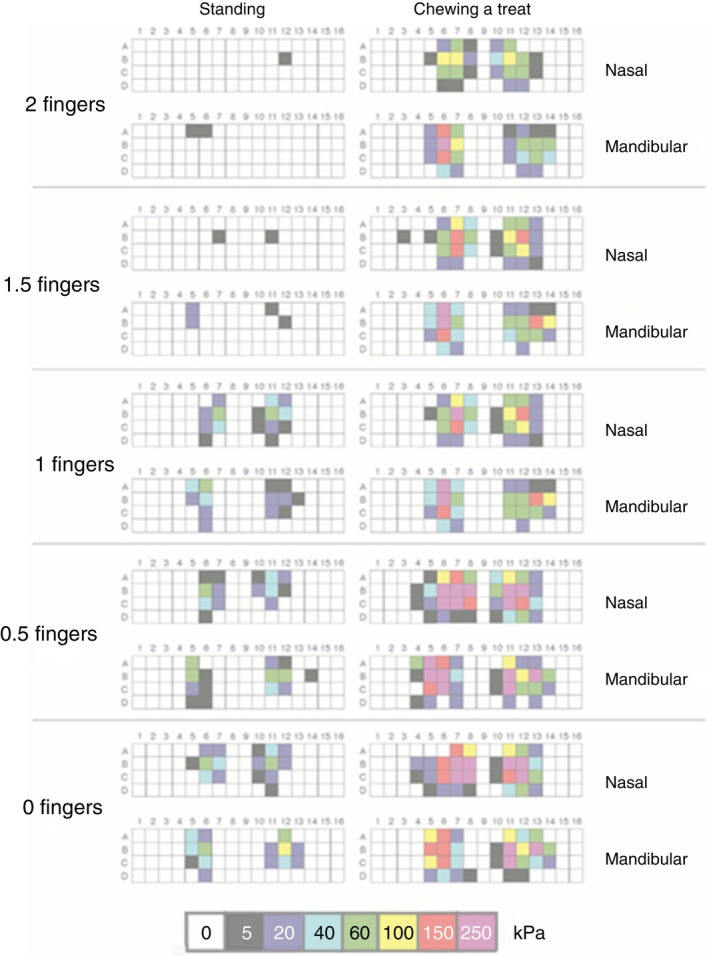
Typical pressure scans for the nasal and mandibular mats on one horse recorded around half way through the standing trial (left column) and at maximal total force during the chewing cycle (right column) with the noseband adjusted to five tightness levels (top to bottom: 2.0, 1.5, 1.0, 0.5 and 0.0 finger‐equivalents).

Pressure scans were captured around half way through the standing force–time trace. The force–time graph shows consistently low force values while the horse was standing. When the horse was standing with the noseband adjusted to 2.0 or 1.5 finger‐equivalents, a few cells showed low pressures (≤40 kPa). The loaded area increased with noseband tightness but, except for one cell in the 0 finger‐equivalents scan, pressure values were less than 100 kPa (Figure 4, left).

When the horse chewed a treat, pressure scans showed that pressure magnitude increased with tightness of the noseband. Pressures up to 250 kPa were recorded on both the nasal and mandibular mats (Figure 4, right). There is an unloaded area in the middle of the nasal scans that represents a recessed area mid‐dorsally at the inter‐nasal suture that is 2–4 cm wide on most scans. The unloaded area in the middle of the mandibular scans is the inter‐mandibular space which is 3–4 cm wide.

### 3.4 Eye temperature

In standing horses, eye temperature did not change with noseband tightness or between the start and end of the trial. When chewing a treat, there were no significant differences in eye temperature between the time of noseband tightening (33.0° ± 1.0) and the end of the trial (32.9° ± 1.0, *p* = 0.03).

### 3.5 Blink rate

Blink rate did not change with increasing noseband tightness during standing (2 finger‐equivalents: 14.8 ± 3.5 blinks/min; 0 finger‐equivalents: 12.4 ± 3.4 blinks/min; *p* = 0.157) or chewing (2 finger‐equivalents: 12.8 ± 4.0 blinks/min; 0 finger‐equivalents: 11.6 ± 3.9 blinks/min; *p* > 0.9).

## 4 DISCUSSION

The total force and pressure distribution beneath a cavesson noseband adjusted to five tightness levels were measured during standing and chewing a treat. The first hypothesis was supported by increases in sub‐noseband force and pressure with noseband tightness but the absence of increases in eye temperature or blink rate did not support the second hypothesis. The results provide quantitative information describing the location and magnitude of pressure on the horse's face that may be applied in regulating noseband adjustment with rules that are fair, safe, and effective in fulfilling the function of the noseband and supporting equine welfare.

A correctly adjusted noseband stabilises the bridle, acts as a training tool by immediate reduction of high pressure levels on the face when the mouth is closed, and promotes safety by aiding in preventing the horse from evading the action of the bit. The cavesson noseband is perceived as kind and comfortable^14^ but excessive tightening may increase the likelihood of oral lesions.^11^

It has previously been suggested that there may be an association between bridle fit and oral lesions.^15^ Oral lesions or blood were seen at the oral commissures in 9.2% of horses competing in dressage, showjumping, eventing and endurance. A higher level of competition was associated with more oral lesions but there was no difference between bit types or bitless bridles.^1^ The role of the noseband in the aetiology of oral lesions is less clear. Although a looser upper noseband has been associated with a reduced risk of oral lesions, the absence of a cavesson noseband increased the risk of lesions at the commissures of the lips 2.39 times compared with the loosest noseband.^1^ On the one hand, the presence of a noseband appears beneficial, perhaps by limiting oral movements and behaviours that may play a role in the development of lesions, but conversely, removal of the noseband does not prevent oral lesions. Furthermore, the noseband does not act in isolation; there are undoubtedly interactions between the noseband, the bit, the teeth and the tongue that will require a multi‐modal research approach to fully evaluate its impact.

Traditionally, noseband laxity has been regarded as sufficient when two human fingers in an unspecified orientation can be inserted between some part of the noseband and the horse's face.^16^ This metric appears inadequate for several reasons including the lack of evidence‐based data to support two fingers as the appropriate level of noseband laxity and human finger dimensions are quite variable.^7^ A transverse section through the horse's nose 1–4 cm rostral to the termination of the facial crest shows that the noseband is supported by bony prominences dorsally (nasal bones), ventrally (mandibular rami), and laterally over the premolar teeth as illustrated in a previous study.^2^ When the noseband is adjusted to 0.0 finger‐equivalents in a standing horse, it touches the skin of the horse's face in the areas of the bony prominences as shown by the standing pressure scans (Figure 4). Thus, a noseband adjusted to 0.0 finger‐equivalents in a horse standing with loose reins is associated with low sub‐noseband pressures. When the noseband is forcibly adjusted tighter than 0.0 finger‐equivalents, compressive forces on the skin, subcutaneous tissues and underlying bones increase markedly. The reason for adjusting the noseband tighter than 0.0 finger‐equivalents may be to prevent the horse from opening its mouth in order to resist the bit or to sensitise the mouth to the action of the bit.^17^, ^18^ There are currently no data describing the effects of nosebands adjusted more tightly than in this study or their role in the development of oral lesions.

In the study reported here, neither eye temperature nor blink rate increased even for the tightest noseband adjustment suggesting that these horses were not experiencing discomfort.^5^, ^6^ These findings contradict a previous report in which young horses that were naïve to wearing a double bridle and Swedish noseband showed a significant increase in eye temperature as the noseband was tightened together with a decrease in skin temperature.^7^ It was not determined whether the response was triggered by the novel equipment or by noseband tightness.

**FIGURE 5:**
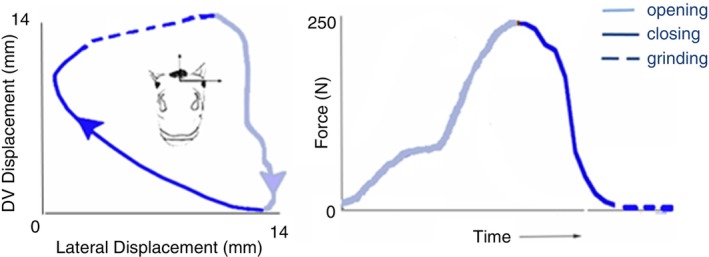
The chewing cycle. Left: rostral view in a transverse plane showing the dorsoventral (DV) and lateral displacements of a virtual midline marker between the mandibular fourth cheek teeth in a horse chewing pelleted feed on the right side.^21^ Right: force–time graph of nasal force during one chewing cycle from the current study. Corresponding phases of the chewing cycle are represented by the same colour and line style.

Movement of the TMJ is important for ingestion of food and balanced tooth wear in the horse, so restriction could have potential problems for joint wear or dental structures.^19^ The TMJ allows 3‐dimensional rotations and translations. The largest rotations are in pitch and yaw (>3°) (Figure 5, left) which open and close the mouth and move the mandibles left to right, respectively. Roll rotation is <1° due to its motion being limited by apposition of the joint surfaces on the opposite side. There is also considerable translational motion in the rostrocaudal (~10 mm), laterolateral (~6 mm) and dorsoventral (~3 mm) directions.^20^ Interactions between rotational and translational motion produce multidirectional movements of the mandible not simply opening and closing of the mouth.

Chewing is a cyclic movement consisting of opening, closing and grinding strokes.^20^ The cycle begins with the jaws closed which coincides with minimal noseband force. As the jaws separate during the opening stroke, noseband force increases then decreases in the following closing stroke. During the grinding stroke, the mandibles move laterally with the cheek teeth in occlusal contact (Figure 5).

A treat was offered to assess the effect of noseband tightness on the horse's ability and willingness to open the mouth widely enough to ingest and chew a treat measuring 4.5 × 1.7 × 1.7 cm. Even at 0.0 finger‐equivalents, all horses willingly ingested and ate the treat without increases in eye temperature or blink rate. We conclude that horses passed the test of being able to eat a treat when wearing a noseband that is adjusted to touch the face. This indicates that some behaviours may be less affected by the noseband than has been assumed in the past.^7^ Based on the data reported here, it is not possible to determine whether chewing amplitude changes, but there was no alteration in chewing frequency with noseband tightness.

One of the salient findings of this study was the horses' willingness to accept considerably higher force (~100 N) and pressure (>40 kPa) during chewing than they experience during ridden exercise.^22^ The pre‐frontal cortex plans behaviours based on previous experiences and a centralised evaluation system recognises the behaviours as pleasurable or stressful.^23^ Animals can learn to control stressful events with top‐down control from the pre‐frontal cortex acting to inhibit the default behaviour.^24^ Food motivation is known to differ between horses but, within an individual, it is consistent across different situations.^25^ Apparently, the anticipated pleasure of eating the treat over‐rode any perceived discomfort. Based on the absence of increases in eye temperature or blink rate, it seems unlikely that these horses found the pressure uncomfortable.

A modelling study^2^ based on anatomical information estimated that noseband pressure would be highest over the mandibular rami and lateral aspect of the nasal bones which are the areas of greatest bony curvature. Data from a horse wearing a Swedish noseband incorporating a load cell to measure noseband tension indicated that mean maximal tensile force was ~40 N when chewing grain with the noseband adjusted to 2.0 finger‐equivalents. Tension was highest during ingestion of feed as in the study reported here.^26^

Few studies have measured sub‐noseband pressures and the findings have been quite variable due, not only with regard to tightness, but also to features such as noseband dimensions and padding. The type, calibration and accuracy of the equipment can have a marked effect on the results.^18^

Previous studies^11^, ^12^, ^26^ focussed on nasal bone pressure, but as predicted by the model,^26^ the data presented here showed mandibular pressure was always higher than nasal pressure though the differences were not statistically significant for all conditions (Table 2). In dressage horses trotting over‐ground, peak noseband pressures were reported to be 53.3 kPa on the lateral nasal bones which was reduced by using a foam pad to offload the high pressure areas.^12^ Since even higher pressures are recorded on the mandibles, the authors suggest that consideration be given to the amount and type of padding used, not only over the nasal bones but also over the mandibles. Ideally, the padding material and the overlying leather should be soft, easily deformable, able to dampen the noseband force and should rebound when unloaded.

Wearing a noseband has been compared to the effect of a tourniquet,^13^ which applies continuous high pressure to occlude underlying vessels. The potential for tissue damage beneath a constricting band depends not only on its tightness but also the duration for which pressure is maintained.^27^ In the study presented here, the pressure at the tightness levels tested did not reach vascular occlusive levels required in a tourniquet and, since high noseband pressures are confined to convexities of the bones,^13^ it is unlikely that the noseband compresses vessels or nerves located in the concave areas that the noseband bridges across.^13^ Jaw movements, including the cyclic force pattern during chewing, are less conducive to developing tissue ischaemia than continuous high force.^27^ We conclude that the noseband, even at 0.0 finger‐equivalents, does not act as a tourniquet.

Little is known about how horses perceive pressure applied by the noseband, more specifically the magnitude and duration of pressure that cause discomfort or pain in specific facial areas. Saddles cause back discomfort when peak pressure exceeds 35 kPa.^28^ Dry spots due to sweat gland ischaemia occurred in areas with maximal and mean pressures of 43.4 and 18.1 kPa, respectively, while overt saddle sores were associated with maximal and median pressures of 53.3 and 29.7 kPa. However, it cannot be assumed that the same pressure thresholds apply to different anatomical areas. Facial expressions characteristic of pain^29^ or aversive responses to putting on the tack may be observed when ill‐fitting tack is used.^30^

The technique of choice for identifying pain thresholds objectively is pressure algometry, in which gradually increasing pressure is applied to a specific area of the body until an avoidance response occurs. The corresponding pressure defines the mechanical nociceptive threshold (MNT) which is the minimal pressure associated with a painful sensation. This technique has been reviewed by Haussler^31^ who described it as ‘a repeatable, semi‐objective method that can be used in a wide array of clinical and research applications to assess MNTs in horses’.

The MNT for different regions of the equine axial skeleton ranged from 400 to 1000 kPa with values increasing in a cranial to caudal direction.^32^ The only site tested on the head was the TMJ where the MNT ranged from 500 to 600 kPa.^32^ This is an order of magnitude greater than the pressure over the nasal bones or mandibular rami reported here.

In the absence of data quantifying MNTs at other sites on the horse's face, the human literature may provide useful information. In people, pressure discomfort thresholds (PDT) and pressure pain thresholds (PPT) have been measured to assist in designing wearables for the face. Over the bridge of the nose, it has been reported that mean PDT value was 181.0 kPa and mean PPT value was 237.6 kPa.^33^ These values greatly exceed the maxima beneath the noseband adjusted to 0.0 finger‐equivalents.

Limitations to the study include the inclusion of only eight horses all of which were highly trained. The effects of noseband tightness were tested in a single type of noseband so the width and type of padding were the same throughout. Different tightness levels were tested in the same order from loosest to tightest in all horses as stipulated by the animal care and use committee, which may have allowed horses to habituate to increasing noseband tightness. The maximal tightness was 0.0 finger‐equivalents. It is undoubtedly possible to tighten the noseband beyond 0.0 finger‐equivalents by compression of the underlying soft tissues which would give a better representation of the effects of a tight noseband. The study did not investigate the horse's perception of noseband pressure or threshold levels for the perception of discomfort or pain which are highly relevant to the determination of acceptable noseband tightness levels.

## 5 CONCLUSION

Traditionally, nosebands were regarded as being of sufficient laxity if two fingers could be inserted between the noseband and the horse's face. In horses accustomed to being ridden in a snaffle bridle, neither eye temperature nor blink rate indicated a stress response at any tightness level ≥0.0 finger‐equivalents during standing or chewing a treat. The absence of an aversive reaction to noseband pressure and the willingness to ingest and chew a treat suggests that the maximal force and pressure recorded here were not perceived as uncomfortable or painful by these horses and did not exceed the MNT. This is consistent with human data describing discomfort/pain thresholds over the nose that are considerably higher than the sub‐noseband pressures reported here. These results suggest that the recommendation of using 2 finger‐equivalents as the standard for noseband tightness should be re‐evaluated based on objective data describing physical parameters and welfare.

## FUNDING INFORMATION

This study was supported by the World Horse Welfare, Equestrian Canada, Hartpury University, British Equestrian Federation and The Worshipful Company of Saddlers.

## CONFLICT OF INTEREST STATEMENT

The authors declare no conflicts of interest.

## AUTHOR CONTRIBUTIONS

**Hilary M. Clayton:** Conceptualization; investigation; funding acquisition; writing – original draft; methodology; visualization; writing – review and editing; project administration; data curation; supervision; formal analysis; resources. **Rachel Murray:** Conceptualization; investigation; funding acquisition; writing – review and editing; visualization; methodology; formal analysis; data curation. **Jane M. Williams:** Conceptualization; writing – review and editing; methodology; formal analysis; data curation; project administration; investigation. **Vicki Walker:** Conceptualization; investigation; methodology; writing – review and editing; formal analysis; data curation. **Mark Fisher:** Conceptualization; investigation; methodology; writing – review and editing. **Diane Fisher:** Conceptualization; investigation; methodology; writing – review and editing. **Jane Nixon:** Conceptualization; investigation; writing – review and editing. **Russell Mackechnie‐Guire:** Conceptualization; investigation; writing – original draft; funding acquisition; validation; methodology; visualization; writing – review and editing; supervision; data curation; project administration; formal analysis; resources.

## DATA INTEGRITY STATEMENT

Hilary Clayton, Rachel Murray, Jane Williams, Victoria Walker and Russell MacKechnie‐Guire had full access to all the data in the study and take responsibility for the integrity of the data and the accuracy of data analysis.

## ETHICAL ANIMAL RESEARCH

The study protocols were approved by Hartpury University's Ethics Committee: URN 2021‐105.

## INFORMED CONSENT

Informed, written consent was obtained from riders and horse owners prior to participation.

## Data Availability

The data that support findings of this study are available from the corresponding author upon reasonable request: Open sharing exemption granted by the editor.
